# *LOC689986*, a unique gene showing specific expression in restricted areas of the rodent neocortex

**DOI:** 10.1186/1471-2202-14-68

**Published:** 2013-07-11

**Authors:** Kari M Ersland, Bjarte Håvik, Johanne Egge Rinholm, Vidar Gundersen, Christine Stansberg, Vidar M Steen

**Affiliations:** 1 Dr E. Martens Research Group for Biological Psychiatry, Department of Clinical Science, University of Bergen, Bergen, Norway; 2 Center for Medical Genetics and Molecular Medicine, Laboratory Building, Haukeland University Hospital, Bergen, Norway; 3 KG Jebsen Centre for Psychosis Research, Oslo, Norway; 4 Department of Anatomy and Centre for Molecular Biology and Neuroscience, University of Oslo, Oslo, Norway; 5 Department of Neurology, Oslo University Hospital, Oslo, Norway

**Keywords:** Cerebellum, Conservation, Cortex, Enriched expression, Somatosensory

## Abstract

**Background:**

The neocortex is a highly specialised and complex brain structure, involved in numerous tasks, ranging from processing and interpretation of somatosensory information, to control of motor functions. The normal function linked to distinct neocortical areas might involve control of highly specific gene expression, and in order to identify such regionally enriched genes, we previously analysed the global gene expression in three different cortical regions (frontomedial, temporal and occipital cortex) from the adult rat brain. We identified distinct sets of differentially expressed genes. One of these genes, namely the *hypothetical protein LOC689986* (*LOC689986*), was of particular interest, due to an almost exclusive expression in the temporal cortex.

**Results:**

Detailed analysis of *LOC689986* in the adult rat brain confirmed the expression in confined areas of parieto-temporal cortex, and revealed highly specific expression in layer 4 of the somatosensory cortex, with sharp borders towards the neighbouring motor cortex. In addition, *LOC689986* was found to be translated *in vivo*, and was detected in the somatosensory cortex and in the Purkinje cells of the cerebellar cortex. The protein was present in neuronal dendrites and also in astrocyte cells. Finally, this unique gene is apparently specific for, and highly conserved in, the vertebrate lineage.

**Conclusions:**

In this study, we have partially characterised the highly conserved *LOC689986* gene, which is specific to the vertebrate linage. The gene displays a distinct pattern of expression in layer 4 of the somatosensory cortex, and areas of the parieto-temporal cortex in rodents.

## Background

The neocortex constitutes the largest part of the highly complex mammalian cerebral cortex. It is radially organised into a six-layered structure, characterised by heterogeneous populations of morphologically and connectionally distinct neurons. The neocortex is further subdivided into specific functional domains, based on both cytoarchitecture and chemoarchitecture, input and output projections, and gene expression patterns [1-3].

During embryonic development, the mammalian neocortex is divided into its functional domains through a process termed arealisation. Although not well understood, the underlying mechanisms that control this process, are thought to involve an interplay between genetic regulation intrinsic to the neocortex, and extrinsic influence from thalamic projection neurons [4-6]. Early in development, morphogens are secreted from telencephalic patterning centres, initiating anterior-medial and posterior-lateral gradients of transcription factors in progenitor cells in the cortical ventricular zone [7]. The graded expression of various transcription factors conveys area identity to cortical progenitor cells, and is subsequently conferred to the neuronal progeny making up the cortical plate. Graded expression of transcription factors is also observed in the developing cortical plate, but this is converted into distinct patterns with sharp borders at later stages [3].

In the adult neocortex, specific regions have distinct functional roles, varying from processing of different somatosensory information, to control of motor functioning. There is limited knowledge about the mechanisms that support functional specialisation of the distinct cortical areas in the adult brain. However, differential gene expression patterns in the adult mouse brain seem to be closely related to, and might be determined by, the position of gene expression along the anterior–posterior axis of the neural tube [8]. Furthermore, control of highly region- and layer-specific gene expression has been linked to the mechanisms supporting the morphological- and functional maturation of the postnatal primate neocortex [9].

In order to identify such area-specific genes, we previously examined the global gene expression in the frontomedial- (FMCx), temporal- (TCx) and occipital (OCx) cortices from the adult rat brain. Although the global gene expression in these three cortical areas was highly similar, we were able to identify distinct sets of genes displaying regional enrichment [10,11]. Among these was a so far unannotated gene, the *hypothetical protein LOC689986* (*LOC689986*), displaying a highly restricted gene expression in the TCx. Based on the assumption that genes highly or specifically expressed within a certain region or organ are likely to reflect its functional specialisation [9,10,12,13], we hypothesised that this gene might have an important role in the TCx.

In this study we have examined *LOC689986* with regard to spatial differential mRNA expression, in addition to protein expression analysis. We also explored the evolutionary conservation and genetic synteny of this unannotated gene. Finally, we investigated the possible functional roles of LOC689986 by various bioinformatics approaches and also by yeast-2-hybrid screens.

## Methods

### Animals and tissue dissection

All animal experiments were approved by and carried out in accordance with the guidelines from the Norwegian Committee for Experiments on Animals (“Forsøksdyrutvalget”, reference number: 20102702 and 2113555). Care was taken to ensure minimal suffering of the animals at all stages of the experiments.

Adult female outbred Sprague–Dawley and male Wistar rats (Mollegaard, Denmark), with body weight of approximately 250 g, were housed for one week before conducting the experiments. Inbred C57BL/6 mice were housed for 5, 10 or 30 days after birth (P5, P10 or P30, respectively), before sacrifice. Rats were anesthetised by isoflurane gas (Isoba vet; Schering-Plough, Denmark) and sacrificed by decapitation. Brain- and non-central nervous system (non-CNS) tissue samples for gene and protein expression analysis were dissected and immediately frozen on dry ice. Cortical tissue samples were extracted from a matrix of side-by-side areas of the adult rat neocortex, covering the occipital-, temporal- and parietal lobe (as depicted in Additional file 1). The area corresponding to the primary auditory cortex was first identified, and subsequently used as a starting point for the dissection of consecutive samples. The whole neocortex (left hemisphere) was isolated, and a total of 25 samples were extracted. Each tissue sample measured approximately 2x2 mm and was dissected from corresponding neocortical areas from six individual rats. All tissue samples were stored at -80°C. For *in situ* RNA hybridisation and immunohistochemistry analysis, rats and mice were first anesthetised by isoflurane gas, followed by intraperitoneal (i.p.) injection of pentobarbital and transcardiac perfusion with 9 mg/ml NaCl and 4% (w/v) paraformaldehyde/PBS. Fixated brains were placed in PBS, soaked in 30% (w/v) sucrose and embedded in Tissue-Tech O.C.T. compound (Sakura Finetek, USA). The embedded brains were frozen on dry ice and stored at -80°C. For pre-embedding electron microscopic immunocytochemistry, rats were anesthetised with pentobarbital (100 mg/kg, i.p.) before fixation through transcardiac perfusion with a solution of 4% formaldehyde in 0.1 M sodium phosphate buffer, pH 7.4 (PB) (50 ml/min for 15 min). The fixed brains were stored in the fixative diluted 1:10 in PB at 4°C.

### RNA purification, cDNA synthesis and gene expression analysis

The tissue samples from rat were homogenised using a Beadmill TissueLyser (Qiagen, Germany), and total RNA was purified from homogenised samples using the ABI PRISM™ 6100 Nucleic Acid PrepStation (Applied Biosystems, USA). The NanoDrop^®^ ND-1000 spectrophotometer (Nanodrop Technologies, USA) was used to measure the RNA quantity and quality. 20 ng total RNA from each sample was reverse transcribed to cDNA using the High Capacity cDNA Reverse Transcription Kit (Applied Biosystems). Total RNA from human brain tissues (i.e. fetal and adult brain, frontal-, temporal- and occipital pole, hippocampus, medulla and cerebellum) was obtained from Clontech (USA). Quantitative real-time PCR (qRT-PCR) was conducted using the ABI Prism 7900HT sequence detector system (Applied Biosystems). The samples were run in triplicates, as previously described [14], and the comparative Ct method [15] was used to determine the relative gene expression levels. The expression level of *hypothetical protein LOC689986* (*LOC689986*, [GenBank: NM_001109563.1]) and the human orthologous gene *Chromosome 1 open reading frame 146* (*C1orf146*, [GenBank: NM_001012425.1]) was measured using TaqMan^®^ Assay probes (Product id: Rn01765992_m1 and Hs00415950_m1, rat and human, respectively) (Applied Biosystems). The expression levels were normalised relative to the endogenous controls *acidic ribosomal phosphoprotein P0* (*Arbp*) and/or *β*-*actin* (*Actb*).

In addition, the Tissue Gene Expression Database (Human Body Map, Applied Biosystems), consisting of 32 different human tissue samples (http://www.ncbi.nlm.nih.gov/projects/geo/query/acc.cgi?acc=GSE7905), was mined in order to screen for expression of the human orthologous gene.

### Cloning and generation of eukaryotic expression vectors

cDNA generated from an adult rat temporal cortex sample was used as template to amplify the full length *LOC689986* transcript; forward primer sequence: 5′-ACAGCCACCCACCCCACA, reverse primer sequence: 5′-GTGTTCCTCTGCAGGAATAGC. The amplified gene was cloned into the pCR^®^II-TOPO^®^ vector (Invitrogen, USA). To generate a vector encoding C-terminally V5-tagged LOC689986, the gene was amplified from the above described vector and ligated into the pcDNA™3.1⁄V5-His A vector (Invitrogen) via its *BamHI*/*ApaI* sites. To generate vectors encoding C- or N-terminally YFP, the gene was amplified from the pCR^®^II-TOPO^®^ vector and ligated into the pEYFP-C1 or pEYFP-N1 vector (Clontech, USA) via its *EcoRI*/*BamHI* or *NheI*/*BamHI* sites, respectively.

### Probe preparation and *in situ* RNA hybridisation

Antisense and sense riboprobes were generated by T7 and SP6 transcription from linearised plasmid (*LOC689986* gene cloned into the pCR^®^II-TOPO^®^ vector) in the presence of digoxigenin labelling mix (Roche, Switzerland). 30 μm thick coronal cryosections were cut through the whole adult rat brain, using a Leica CM3050 cryostat, and floating sections were treated as previously described [16]. In short, sections were permeabilised with Proteinase K (20 μg/ml), fixated in 4% (w/v) paraformaldehyde/PBS, treated with 25% (v/v) acetic anhydride in 0.1 M TEA (pH 8), following application of riboprobes in hybridisation buffer to the sections. Sense riboprobes were included in all experiments as a negative control. The hybridisation reaction was left for at least 16 hours at 60°C, and the sections were then washed thoroughly prior to RNase A treatment (20 μg/ml). Alkaline coupled anti-digoxigenin antibody was applied (diluted 1:2000) and visualisation was achieved by using NBT/BCIP chromogen substrates (Roche).

### Production of rabbit anti-LOC689986 peptide antibody

A polyclonal peptide antibody, targeting a C-terminal epitope with amino acid sequence: IEQSPVWRTLQK, was generated in rabbits by 21st Century Biochemicals (Marlboro, MA, USA). Polyclonal serum was affinity purified and the peptide antibody was subsequently used in western blot- and immunohistochemistry analysis.

### Protein determination, gel electrophoresis and western blot analysis

Homogenised tissue samples from rat and cell lysates from transiently transfected HeLa cells were prepared in RIPA Triton X-100 buffer (150 mM NaCl, 1% (v/v) Triton X-100, 0.5% (w/v) sodium deoxycholate, 0.1% SDS (w/v) and 50 mM Tris/HCl pH 8.0). Protein concentrations were determined using the *DC* Protein Assay Kit (Bio-Rad, USA). Polyacrylamide gel electrophoresis and immunoblotting were performed according to the manufacturer’s instructions using NuPAGE^®^Bis-Tris pre-cast gels 10% (Invitrogen). Primary antibodies used were: rabbit anti-LOC689986 peptide antibody (21st Century Biochemicals), mouse anti-V5 (Invitrogen), goat anti-Gapdh and goat anti-Actin (Santa Cruz Biotechnology). Secondary antibodies used were: donkey anti-mouse IgG-HRP, donkey anti-rabbit IgG-HRP and donkey anti-goat IgG-HRP (Santa Cruz Biotechnology). Enhanced chemiluminescence (GE Healthcare, United Kingdom) was used for detection, and equal protein loading was examined by either Gapdh or Actin immunodetection. Pre-absorption controls were included by incubating the anti-LOC689986 antibody with the peptide used to generate the antibody (1 hour at room temperature) prior to use.

### Immunohistochemistry analysis

20 μm sagittal cryosections were cut from embedded mouse brains using a Leica CM3050 cryostat, collected and thaw-mounted onto SuperFrost^®^ Plus slides (Thermo Fisher Scientific, USA). Sections were dried for 30 min at 37°C and rinsed briefly in PBS. After blocking in 5% (w/v) bovine serum albumin (Sigma-Aldrich, USA) and 0.2% (v/v) Triton X-100 in antibody buffer (150 mM NaCl, 50 mM Tris–HCl, 1% (w/v) bovine serum albumin, 100 mM L-Lysine, 0.04% (w/v) Sodium Azide) for 1 hour at room temperature, primary antibodies were applied and the slides were incubated at 4°C overnight. Primary antibodies used were: rabbit anti-LOC689986 peptide antibody (diluted 1:200, 21st Century Biochemicals) and mouse anti-200 kD Neurofilament Heavy Monoclonal antibody (diluted 1:500, Abcam, United Kingdom). Slides were washed three times in PBS, and incubated for 2 hours at room temperature in highly cross-absorbed fluorescent-conjugated secondary antibodies (Invitrogen); Alexa Fluor^®^ 488 goat anti-rabbit IgG (diluted 1:1000) and Alexa Fluor^®^ 594 goat anti-mouse IgG (diluted 1:1000). Nuclei were stained using DAPI. Slides were mounted using Vectashield mounting medium (Vector Labs, USA) and fluorescent images were obtained by a Zeiss LSM 510 META (Zeiss, Germany) or Leica TCS SP2 AOBS (Leica Microsystems, Germany) confocal microscope.

### Cell culturing, transient transfection and immunocytochemistry

Human HeLa cells (ATCC-LGC, USA) were cultivated in Eagle’s Minimum Essential Medium supplemented with 10% (v/v) fetal bovine serum and penicillin/streptomycin. Cells were transiently transfected for 24–48 hours using Lipofectamine 2000 Transfection Reagent (Invitrogen) according to the recommendations of the manufacturer. Transiently transfected cells were grown on cover slips and fixated using 4% (w/v) paraformaldehyde/PBS for 45 min. Cells were permeabilised for 15 min by subjecting them to 0.5% (v/v) Triton X-100 in PBS treatment. Detection of recombinant protein was achieved either directly (transient transfection using vectors encoding either a C- or N-terminal YFP tagged recombinant protein), or by using mouse anti-V5 primary antibody (diluted 1:1000, Invitrogen) and Alexa Fluor^®^ 594 goat anti-mouse IgG (diluted 1:1000, Invitrogen) secondary antibody. Nuclei were stained with DAPI. Images were obtained by using a Leica TCS SP2 AOBS confocal microscope (Leica Microsystems).

### Pre-embedding electron microscopic immunocytochemistry

Frontal sections (50 μm) of two fixed rat brains were cut on a vibratome, and labelled free-floating with the rabbit anti-LOC689986 peptide antibody (diluted 1:50, 21st Century Biochemicals) according to a three-layer immunoperoxidase method, in which the antigen-antibody binding is visualised by an electron dense diaminobenzidine reaction product. To preserve the ultrastructural morphology, the sections were processed without detergent. Samples containing layer 1–3 of somatosensory cortices were dissected out of the stained sections, dehydrated and embedded in Durcupan ACM Fluka (Sigma-Aldrich). Then ultrathin sections (gold colour) were cut on 300 mesh nickel grids. The ultrathin sections were viewed in a Tecnai 12 electron microscope and electron micrographs at x43,000 magnification were taken in layer 2 at both surfaces of the sections.

### Protein-protein interaction analysis

A yeast-2-hybrid (Y2H) screen was performed by using the full *LOC689986* open reading frame as bait to screen both adult and embryonic (E10.5-E12.5) mouse brain libraries. The analysis was performed by using the ULTImate Y2H™ screen at Hybrigenics Services (France, http://www.hybrigenics-services.com/). A total of 88.47 and 65.1 million interactions were analysed in the embryonic and adult mouse brain libraries, respectively. Hybrigenics assigns a statistical confidence score, the Predicted Biological Score (PBS^®^), to each interaction. In short, interacting proteins are ranked according to both local and global technical parameters to compute the final score. The PBS^®^ is computed as an expected value (e-value), ranging from 0 (specific interaction) to 1 (probable artefact). For practical purposes these scores are divided into four categories, ranging from A (close to 0, very high confidence in the interaction) to D (close to 1, very low confidence in the interaction) [17]. More details regarding the scoring and ranking of the protein-protein interactions can be found at the Hybrigenics homepage (http://www.hybrigenics-services.com/contents/our-services/discover/ultimate-y2h-2/ultimate-deliverables).

### Web-based bioinformatic tools

Genomic searches were performed using the UCSC Genome Bioinformatics database and the NCBI database. BlastView from the Ensembl Genome Browser (release 63) [18] was used to search for homologous sequences in the rat genome database. BlastView was also applied to search for orthologous sequences in both vertebrate and invertebrate species (i.e. *Caenorhabditis elegans* and *Drosophila melanogaster*), as well as a yeast genome database (i.e. *Saccharomyces cerevisiae*). All searches were conducted using BLAT default settings. Nucleotide sequences were retrieved from the NCBI and UCSC databases. Multiple sequence alignments were performed using ClustalW2 from EMBL-EBI applying default settings (http://www.ebi.ac.uk/Tools/services/web/toolform.ebi?tool=clustalw2) [19,20]. The sequence conservation between various vertebrate species was analysed by exploring the UCSC database. Genetic synteny analysis was performed by exploring the Genomicus v64.01 database, using default settings (http://www.dyogen.ens.fr/genomicus-64.01/cgi-bin/search.pl). In order to examine whether LOC689986 belongs to known protein families or contains known domains, regions or sites, InterProScan Sequence Search from EMBL-EBI was used (http://www.ebi.ac.uk/Tools/pfa/iprscan/) [21]. Prediction of signal peptide cleavage sites was performed by the SignalP 3.0 Server from the Center for Biological Sequence Analysis (http://www.cbs.dtu.dk/services/SignalP/) [22-24]. MyHits was explored to examine potential motifs and post translational modifications of the predicted protein (http://myhits.isb-sib.ch/cgi-bin/motif_scan). Finally, we used the PSIPRED Protein Structure Prediction Server from the UCL-CS Bioinformatics (http://bioinf.cs.ucl.ac.uk/threader/), to analyse the predicted LOC689986 amino acid sequence.

## Results

### *LOC689986* shows distinct gene expression patterns in the adult rat neocortex

**Figure 1:**
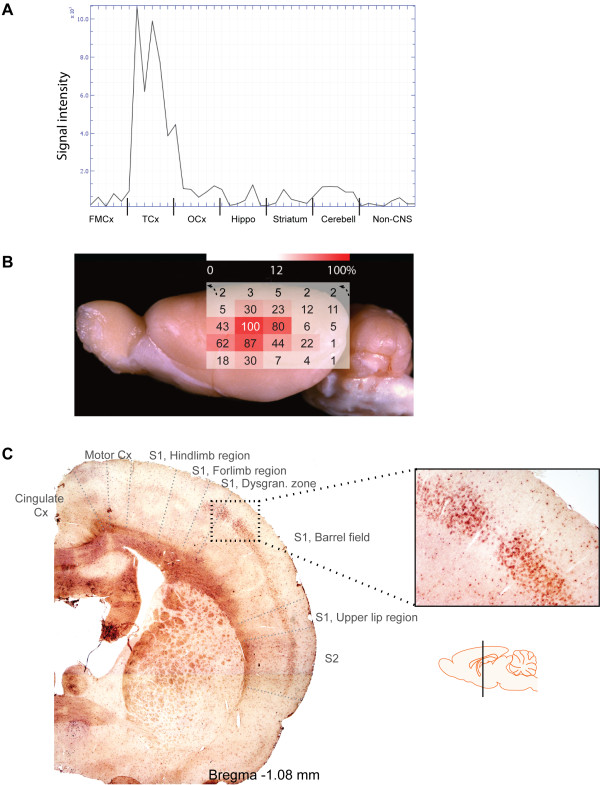
**Overview of *LOC689986 *gene expression patterns in the adult rat brain. A**. Microarray gene expression profile illustrating the differential *LOC689986* expression in areas of the adult rat brain and non-CNS tissues (kidney, liver and spleen). A total of six brain samples from three individual rats, both left and right hemispheres, in addition to non-CNS samples are indicated on the x-axis. The normalised signal intensity is indicated on the y-axis. FMCx: frontomedial cortex, TCx: temporal cortex, OCx: occipital cortex, Hippo: hippocampus, Cerebell: cerebellum. The gene expression profile was generated based on microarray data obtained from [10,11]. **B**. The *LOC689986* average relative gene expression in tissue samples from 25 consecutive areas of the adult rat neocortex, covering the temporal- parietal- and occipital lobes (left hemisphere, Additional file 1). Corresponding samples from six individual adult rats were analysed. *Actb* was used as an endogenous control to normalise the expression levels, and the average relative gene expression in the 25 neocortical samples was transformed into percentage values, based on the region showing the highest gene expression (set to 100%, all other regions were relative to this). The gene expression is indicated by a heat-map superimposed on the rat brain (red: high expression, white: low expression). The uppermost row in the heat-map (black arrows indicating the direction) represents samples from the cingulate cortex. The figure is based upon a picture of the adult rat brain (lateral view, image courtesy of Adam C. Puche) and acquired from The Olfactory Image Archive. **C**. *In situ* RNA hybridisation analysis on coronal sections of the adult rat brain, illustrating the mRNA expression of *LOC689986* in layer 4 of the primary and secondary SCx. The approximate location of the different neocortical areas is indicated [25]. The schematic drawing of the brain which indicates the area analysed was obtained from http://www.motifolio.com.

The rat *hypothetical protein LOC689986* (*LOC689986*) was identified from a previous microarray study of the global gene expression in the FMCx, TCx, OCx, striatum, hippocampus, cerebellum and three non-CNS samples (kidney, liver and spleen) [10,11]. *LOC689986* displayed an almost exclusive expression in samples from the TCx (Figure 1A), with only weak, or no expression in the other brain regions as well as in the three non-CNS tissues examined.

Regionally enriched genes may imply functional specialisation, and in order to analyse the *LOC689986* gene expression in further detail, we extracted consecutive tissue samples from a matrix of 25 areas of the adult rat neocortex, covering the occipital-, temporal- and parietal lobe (Additional file 1; for details see the Methods section). We analysed the gene expression level by qRT-PCR in corresponding samples from six individual rats, and the average relative gene expression was transformed into percentage values based on the region showing the highest gene expression. The gene expression is indicated by a heat-map superimposed on a lateral view of the adult rat brain (Figure 1B; see also Additional files 1 and 2). The strongest expression was found in an area of the primary somatosensory cortex (SCx), namely in the caudal ventral part of the parietal cortex area 1 (Par1, 100%; Figure 1B). Strong expression was also observed in the dorsal part of parietal cortex area 2 in the secondary SCx (Par2, 62-87%). In addition, the dorsal part of the TCx area 1 (Te1, 80%) and the TCx area 3 (Te3R, 80%), corresponding to the TCx area analysed in the initial microarray study, displayed high transcription levels. The gene was also expressed in the neighbouring areas, although to a markedly lower extent, creating a steeply declining gradient. Only negligible gene expression was detected in the samples from the occipital lobe, in agreement with the findings from the initial microarray study.

Next, we analysed coronal sections of the whole adult rat brain by *in situ* RNA hybridisation. The strongest gene expression was found in areas of the SCx, with a rather abrupt absence in the neighbouring motor cortex (MCx) (Figure 1C, Additional file 3). Interestingly, *LOC689986* was specifically expressed in cortical layer 4 in the primary and secondary SCx, including the barrel field. The sense probe (negative control) generated no detectable signal (data not shown).

We also analysed the gene expression of the human orthologous gene *C1orf146*, by qRT-PCR in eight human tissue samples (i.e. whole fetal and adult brain, frontal-, temporal- and occipital pole, hippocampus, medulla and cerebellum). The highest relative gene expression was confined to the frontal pole sample, although *C1orf146* expression was also detected in samples from the medulla, hippocampus and cerebellum (Additional file 4). The expression pattern of *C1orf146* was further explored in microarray data from 32 different human tissues from the Tissue Gene Expression Database (Human Body Map, Applied Biosystems), which included fetal and adult brain. The gene showed strong expression only in samples from testis, and very weak, or no expression in the CNS and the other non-CNS samples (Additional file 5).

### *LOC689986* is highly conserved in vertebrate species

**Figure 2:**
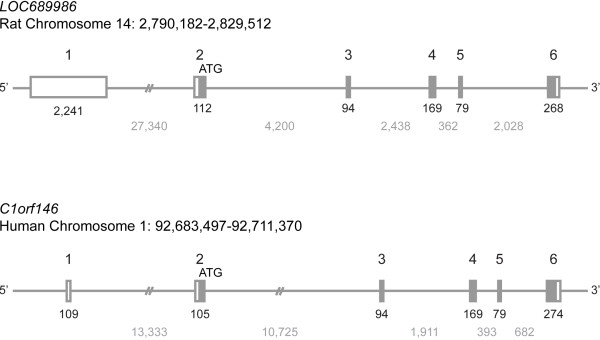
**Genomic organisation of the rat *LOC689986 *gene and the human ortholog *C1orf146*. ***LOC689986* is localised on chromosome 14 in rat, while the human ortholog (*C1orf146*) is localised on chromosome 1. Chromosomal positions are indicated. Both the rat and human gene consists of six exons, with the predicted start codon localised in exon 2. Open box: UTR regions. Filled box: coding region. Exon number is indicated above the boxes. The number of base pairs constituting the exons and introns are visualised in black and grey, respectively, underneath the corresponding genomic structure.

The *LOC689986* gene is quite small and consists of 6 exons located on chromosome 14p22 in the rat genome (Figure 2). The predicted start codon is located in the second exon. The estimated transcription length is 2,963 base pairs, with an open reading frame encoding a predicted protein of 185 amino acids, with a calculated molecular mass of 20.7 kDa. The human orthologous gene showed a similar genomic organisation to the rat gene (Figure 2), consisting of 6 exons with the predicted start codon localised to the second exon.

**Figure 3:**
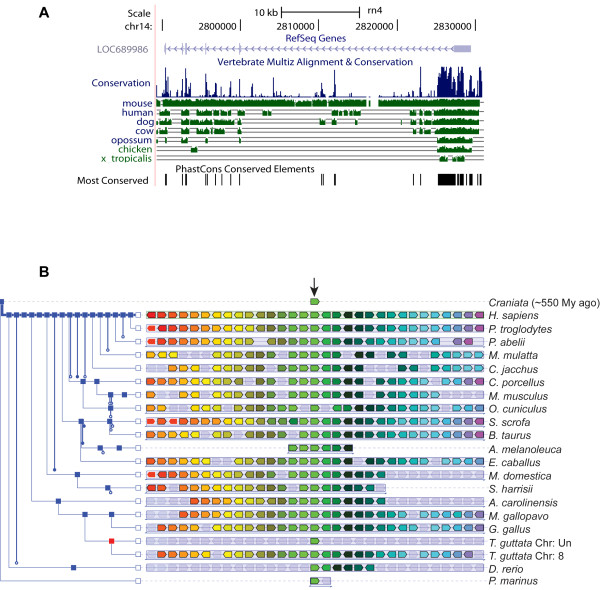
**Conservation of *LOC689986 *orthologs in the vertebrate lineage. A**. Analysis of the sequence conservation of *LOC689986* in various vertebrate species, generated by the UCSC browser (http://genome.ucsc.edu/). **B**. Illustration of the genetic synteny in various vertebrate species, generated by the Genomicus v64.01 database. The human orthologous gene (*C1orf146*) was used as a reference gene. The orthologous genes from various vertebrates are illustrated as a light green box in the centre of the image (indicated by an arrow). The surrounding genes that are in common between the vertebrate species are illustrated by coloured boxes (ranging from red to purple from left to right, respectively). Shaded boxes illustrate genes that are not derived from the same ancestral genomic region. The species names are listed to the right. A phylogenetic tree illustrating the evolutionary relationship between the various vertebrate species was also generated by the Genomicus v64.01 database (shown to the left). Blue squares represent speciation nodes, while the red square represents a duplication node.

By BLAT searches we identified orthologous genes in a wide range of vertebrate species. In contrast, no orthologues were detected in invertebrates and yeast (i.e. *Caenorhabditis elegans*, *Drosophila melanogaster* and *Saccharomyces cerevisiae* databases). We also analysed the *LOC689986* genome sequences from various vertebrate species and found that the gene is highly conserved (Figure 3A). The highest conservation was observed in mammalian species, while the most divergent sequences were found in chicken and frog (Figure 3A). In addition, analysis of the region surrounding the gene revealed that it is located in a large synteny block in various vertebrate species (Figure 3B).

### LOC689986 protein expression in the adult rat brain

**Figure 4:**
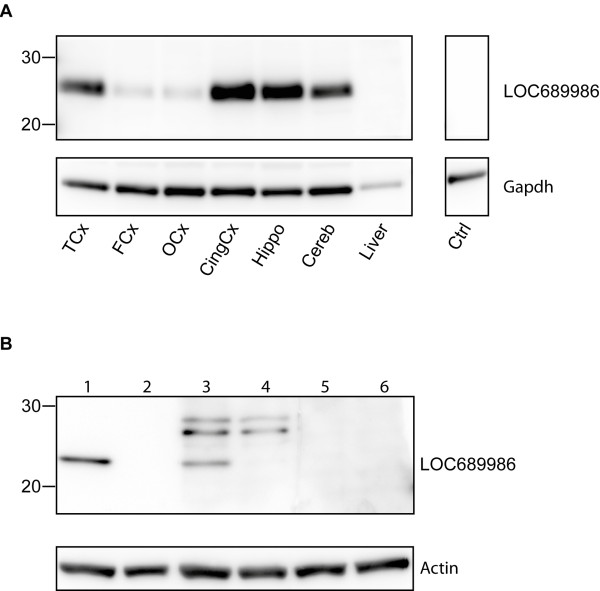
**LOC689986 protein expression analysis. A**. The rabbit anti-LOC689986 antibody recognised a protein of approximately 25 kDa in tissue lysates from the temporal cortex, cingulate cortex, hippocampus and cerebellum. Gapdh immunodetection was used to examine equal protein loading. Ctrl: tissue lysate from a temporal cortex sample not subjected to anti-LOC689986 antibody. **B**. A protein of approximately 23 kDa was detected in transiently transfected HeLa cells, expressing a V5 tagged recombinant LOC689986 protein, using both an anti-V5 antibody (lane 1) and the anti-LOC689986 peptide antibody (lane 3). No recombinant protein was detected in mock-transfected cells (lane 2 and 4). No signal was detected in cell lysates from cells overexpressing the V5 tagged recombinant protein, or mock-transfected cells, when the rabbit anti-LOC689986 antibody was preabsorbed (lane 5 and 6, respectively). Actin immunodetection was used to check for equal protein loading.

To examine whether *LOC689986* was translated *in vivo*, we analysed rat tissue samples from FMCx, TCx, OCx, cingulate cortex, hippocampus, cerebellum and liver. Western blot analysis of tissue lysates, using a custom-made polyclonal peptide antibody, revealed a robust protein band of approximately 25 kDa in the TCx and only very weak expression in FMCx and OCx (Figure 4A). These findings indicate a similar differential expression, at the protein level, as observed from the gene expression data in the initial microarray study. Surprisingly, protein expression could also be detected in samples from the cingulate cortex, hippocampus and cerebellum (Figure 4A), even though mRNA expression was only detected at low levels in these regions (Figure 1A and 1B). In concordance with the transcript analysis, no protein expression of LOC689986 was detected in the tissue sample from liver (Figure 4A). As a control for the specificity of the custom made peptide antibody we included pre-absorption controls. After incubation with pre-absorbed anti-LOC689986 antibody, no protein bands could be detected (data not shown).

The protein detected in tissue lysates by the custom-made peptide antibody had a molecular weight that was approximately 4 kDa higher than the predicted size of LOC689986, which could indicate that the protein had undergone post-translational modifications. We analysed lysates from both transiently transfected HeLa cells over-expressing recombinant LOC689986 tagged by a V5 epitope and mock-transfected cells. The calculated size of the recombinant protein with a V5 tag was approximately 23 kDa. A band of the correct size was detected in cell lysate from cells expressing the recombinant protein using an anti-V5 antibody (Figure 4B, lane 1). In addition, several protein bands were found in the cell lysate from cells over-expressing the recombinant protein, but they were also detected in mock-transfected cells, by using the custom-made anti-LOC689986 peptide antibody (Figure 4B, lane 3 and 4). Moreover, a band of 23 kDa was detected in transiently transfected cells, which could not be detected in the control cells (Figure 4B, lane 3 and 4). Analysis of the cell lysate from transfected- and mock-transfected cells, by using the pre-absorbed peptide antibody, generated no detectable protein bands (Figure 4B, lane 5 and 6). In addition, no protein band of the correct size was detected by western blot analysis of the growth medium of cultured cells, implying that the recombinant protein was not secreted (data not shown).

### The mouse ortholog of rat LOC689986 is expressed in specific areas of the neocortex and cerebellar cortex at three postnatal stages

**Figure 5:**
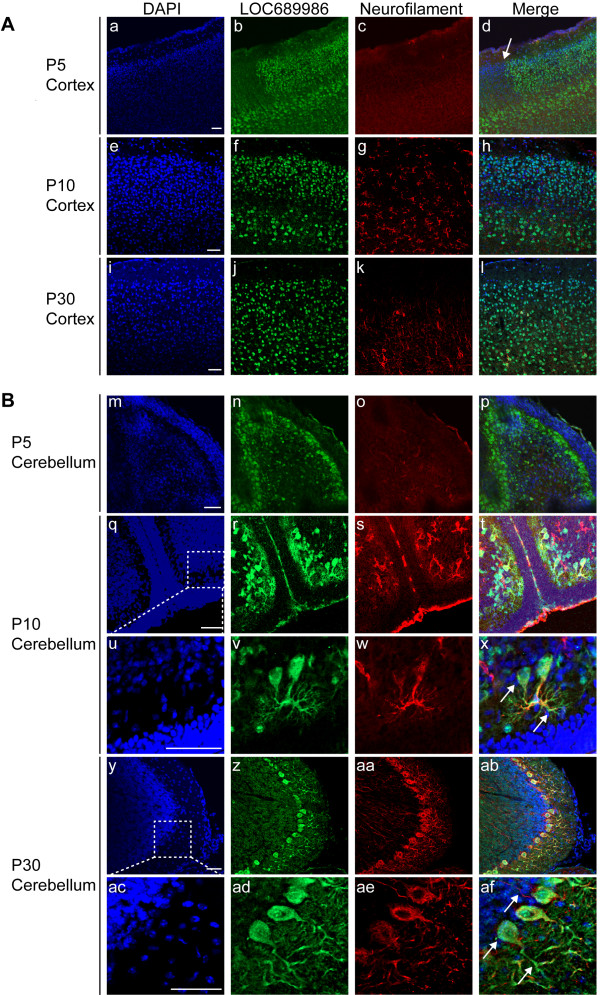
**Immunohistochemistry analysis of the LOC689986 mouse ortholog in the developing and adult mouse brain.** 20 μm sagittal cryosections from mice at three postnatal stages (P5, P10 and P30) were subjected to immunohistochemistry analysis. The fluorescent micrographs depict nuclei (DAPI), expressed LOC689986 mouse ortholog (LOC689986), 200 kD Neurofilament Heavy-chain (Neurofilament) and a merged image. **A**. Expression of the mouse ortholog (1700028K03Rik) in SCx of mice at P5 **(a-d)**, P10 **(e-h)** and P30 **(i-l)**. An abrupt border of protein expression observed between the SCx and the neighbouring MCx at P5 is indicated **(d)**. **B**. Expression of the mouse ortholog (1700028K03Rik) in cerebellum of P5 **(m-p)**, P10 **(q-x)** and P30 **(y-af)** mice. The mouse ortholog (1700028K03Rik) partially overlaps with the neurofilament marker at both P10 **(t** and **x)** and P30 **(ab** and **af)**. The protein is apparently present in the cell body, nucleus and dendrites of Purkinje cells in the cerebellum **(**P10: **t**, and magnification: **x**, P30: **ab**, and magnification: **af)**. *Bar*: 50 μm.

**Figure 6:**
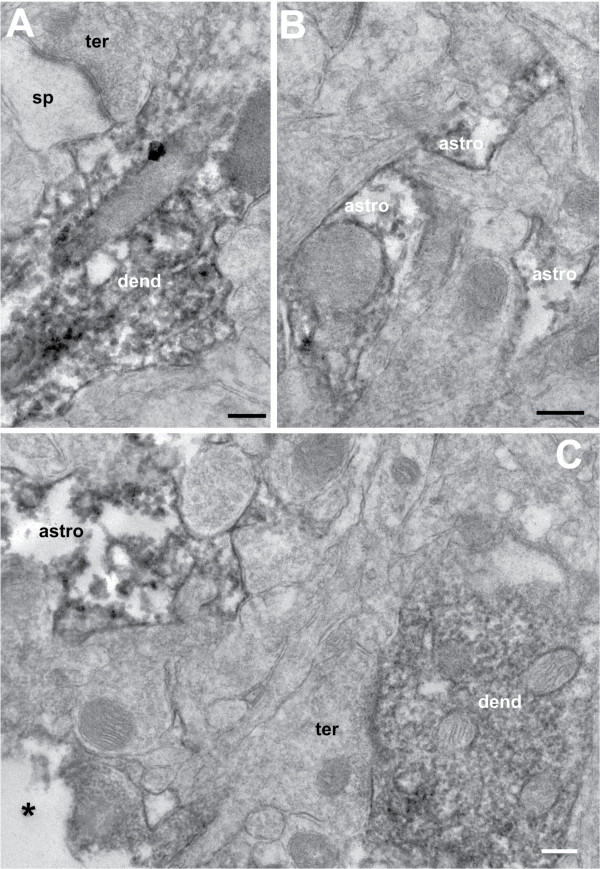
**LOC689986 localises to neuronal dendrites and small astrocytic processes.** LOC689986 localisation in the adult rat somatosensory cortex was analysed by pre-embedding peroxidase immunostaining and electron microscopy **(A**-**C)**. Staining for LOC689986 was present in dendritic shafts (dend) and small astrocytic processes (astro), whereas nerve terminals (ter) and dendritic spines (sp) were unlabelled. *: surface of the section, scale: 100 nm.

The custom-made peptide antibody recognised an epitope that shared 100% sequence identity with the mouse orthologous peptide sequence of rat LOC689986 (official full name of the mouse orthologous gene: *RIKEN cDNA 1700028K03 gene* (*1700028K03Rik*)). We were therefore able to use the anti-LOC689986 peptide antibody to analyse the protein expression in sagittal sections of the mouse brain, by immunohistochemistry at three different postnatal stages (P5, P10 and P30). We found that the protein was expressed in the SCx at P5, P10 and P30 (Figure 5A, a-l). In contrast to the layer specific gene expression observed by *in situ* RNA hybridisation analysis, we were unable to determine any layer specific protein expression in the sagittal sections. At P5, a sharp border of 1700028K03Rik expression could be observed between the SCx and the neighbouring MCx. Strikingly, we also observed strong protein expression in the Purkinje cells in the cerebellar cortex, at all the postnatal stages (Figure 5B, m-af). The protein expression co-localised with the neuronal marker at P10 and P30 in the Purkinje cells (Figure 5B, q-af). However, at P5, the co-localisation was not as clear, possibly reflecting that the Purkinje cells have not fully matured at this stage (Figure 5B, p). Furthermore, 1700028K03Rik protein was detected in the cell body, nucleus and dendrites of the Purkinje cells (Figure 5B, magnification of Purkinje cells at P10 and P30, x and af, respectively). No co-localisation with the neuronal marker was observed in what appeared to be the axons (Figure 5B, af). In addition, we observed protein expression in some areas of FMCx, OCx and hippocampus at P5 (data not shown). Further investigation of the sub-cellular localisation of the LOC689986 protein at the electron microscopic level in the adult rat SCx confirmed that the protein was present in neuronal cell bodies and proximal stem dendrites. We also found labelling in distal dendritic shafts, but there was no sign of LOC689986 signals in dendritic spines (Figure 6A and C). We could not find any evidence of nerve terminal or axonal labelling. LOC689986 also localised to astrocytes (Figure 6B and C, Additional file 6).

### LOC689986 displays nuclear and cytosolic localisation

**Figure 7:**
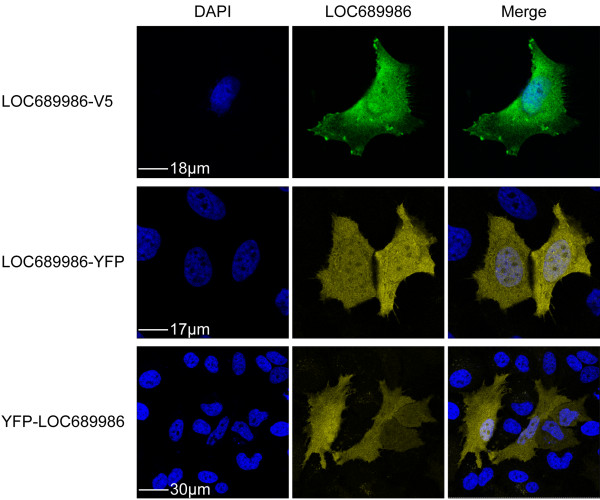
**Cellular localisation of the endogenous and recombinant LOC689986 protein.** Human HeLa cells were transiently transfected with a vector encoding LOC689986 as a V5 tagged protein, or as a C- or N- terminally YFP tagged protein. After 24 hours the cells were subjected to V5- or YFP immunocytochemistry. The recombinant LOC689986 proteins are expressed in both the nucleus and the cytosol. The fluorescent micrographs depict nuclei (DAPI), expressed recombinant LOC689986 (LOC689986) and a merged image. Bars are indicated in the DAPI pictures.

In order to further analyse the cellular localisation of LOC689986, we examined transiently transfected HeLa cells expressing V5-tagged and C- or N-terminal YFP-tagged recombinant proteins. We found a uniform expression of the recombinant proteins, both in the nucleus and cytosol (Figure 7). The localisation of over-expressed V5-tagged protein was also analysed in human neuroblastoma (SH-SY5Y) and glioma (GaMg) cell lines, resulting in similar findings (data not shown).

### LOC689986 could be a transmembrane protein with several potential protein interaction partners

In order to gain insight into potential functional roles of the LOC689986 protein, several web-based bioinformatics tools were utilised. Analysis of the LOC689986 amino acid sequence by InterProScan Sequence Search, confirmed that the predicted protein neither belongs to any known protein families, nor contains known domains or regions with known function. Moreover, no signal peptide cleavage sites were identified by using SignalP 3.0 Server, which could indicate that LOC689986 is a non-secretory protein. Furthermore, no strong evidence for post-translational modifications (i.e. phosphorylation) was found, by MyHits. Finally, we used the PSIPRED Protein Structure Prediction Server from UCL-CS Bioinformatics in order to assess the secondary structure. LOC689986 was predicted to contain 6 α-helices and 6 β-sheets, together with a transmembrane domain (amino-terminus towards the cytosol).

A Y2H screen was performed to search for putative protein-protein interaction partners of the unannotated protein. We used the full length LOC689986 from rat as bait, and screened both adult and embryonic mouse brain libraries. The screen resulted in the identification of five potential binding partners common for the two libraries, namely Chromodomain helicase DNA-binding protein 3 (Chd3), Spectrin beta 2 (Spnb2), SUMO1/sentrin specific peptidase 6 (Senp6), Zinc finger protein 507 (Zfp507) and ElaC homolog 2(E.coli) (Elac2) (Table 1). In addition, two potential interaction partners were identified exclusively in the embryonic mouse brain library, namely Chromodomain helicase DNA-binding protein 4 (Chd4) and Eukaryotic translation initiation factor 4A-1 (Eif4a1) (Table 1). In general, protein-protein interactions displaying the best predicted biological score were observed in the embryonic mouse brain library screen. Here, the most significant interactions were observed for Chd3, Chd4, Spnb2 and Eif4a1. The potential interaction between Senp6 and LOC689986 was listed as an interaction with high confidence in both the embryonic and adult mouse brain libraries.

**Table 1:** Potential LOC689986 protein interaction partners identified by Y2H screens

	**Mouse brain library**	
**Protein**	**Embryonic**	**Adult**
Chromodomain helicase DNA-binding protein 3	+++	+
Chromodomain helicase DNA-binding protein 4	+++	-
Spectrin beta 2	+++	+
Eukaryotic translation initiation factor 4A-1	+++	-
SUMO1/sentrin specific peptidase 6	++	++
Zinc finger protein 507	++	+
ElaC homolog 2(*E*.*coli*)	+	++

The Y2H screens revealed five potential interaction partners common for both the adult and the embryonic mouse brain libraries. Two additional potential binding partners were identified in the embryonic mouse brain library. The potential interaction partners are listed based on the global Predicted Biological Score (PBS^®^). +++: very high confidence in the interaction (PBS^®^: A). ++: high confidence in the interaction (PBS^®^: B), +: good confidence in the interaction (PBS^®^: C), “-”: not detected.

The relative gene expression of the five potential interaction partners, identified from both embryonic and adult brain, was examined by qRT-PCR, using the rat brain samples analysed for *LOC689986* gene expression (Additional file 1). We found that the five genes were expressed in all the cortical samples analysed, with no preference for a specific cortical region (data not shown).

## Discussion

We have previously demonstrated that the global gene expression profile in different neocortical areas (i.e. FMCx, TCx and OCx) of the rat brain is surprisingly similar [10,11]. Despite this, we have identified a distinct set of 65 genes, showing regional enrichment in either the FMCx, TCx or OCx [11]. Among these, *LOC689986*, an unannotated and unique gene displaying an almost exclusive expression in samples from the adult rat TCx, showed the overall strongest regional enrichment observed in the analysis [11]. Since genes that are highly- or specifically expressed within a region or organ are often linked to the specialised functions of an area [9,10,12,13], we hypothesised that *LOC689986* could be important for the normal function in the TCx and nearby areas of the rodent brain.

### *LOC689986* is expressed in the parieto-temporal cortex and cerebellar purkinje cells in rodents

By fine-mapping of the parietal, temporal and occipital cortices of the adult rat, we found that *LOC689986* displayed the overall strongest expression in the primary SCx. High expression was also detected in the secondary SCx. *In situ* RNA hybridisation confirmed this expression pattern, and revealed that the activity was confined to cortical layer 4. Furthermore, the mouse orthologous LOC689986 protein could be detected in the SCx at all postnatal stages analysed. Notably, a sharp border of protein expression could be observed between the SCx and the neighbouring MCx at postnatal stage 5, clearly demonstrating the regional specificity of the protein expression. In rodents, the primary SCx is characterised by distinct barrel fields in cortical layer 4, each corresponding topographically to specific whiskers. Layer 4 receives somatosensory input from ventrobasal nucleus projections (ventroposteriomedial thalamic nucleus), in response to stimuli acquired from the rodent whiskers [26]. It was recently demonstrated that genes exhibiting a layer-specific pattern of expression are more likely to encode proteins that are involved in specialised functions (e.g. synaptic transmission and ion transport), whereas genes that displayed a more uniform pattern of expression were linked to cellular “housekeeping” roles [27]. Interestingly, in a recent study describing the global gene expression in the adult rat barrel cortex, *LOC689986* was found to be one of several up-regulated genes in response to enriched environmental stimulation, linking this gene to experience-dependent plasticity in the rat [28].

We also observed *LOC689986* gene expression in restricted areas of the parieto-temporal cortex corresponding to the primary and secondary auditory cortex (Te1 and Te3R, respectively). These cortical areas are implicated in processing of auditory stimuli and receive signals from the medial geniculate body that terminates in layers III and IV, and in the junction between layer V and VI [29].

Interestingly, expression of the mouse orthologous LOC689986 protein was also detected in the Purkinje cells of the cerebellar cortex in the three postnatal stages analysed. The Purkinje cells are among the largest neuronal cells in the brain, implicated in motor functioning, learning and cognitive abilities. In our initial microarray study, we observed only very low *LOC689986* gene expression within the cerebellum tissue samples. However, these whole tissue samples represented a heterogeneous population of cell types, and the expression specific to a certain cell type would be rather diluted. Since LOC689986 was found to be restricted to the Purkinje cells of the cerebellum, it is possible that the samples analysed by microarray contained a very low concentration of the *LOC689986* mRNA, compared to the total mRNA extracted from the tissue samples, explaining why the gene was not detected at higher levels in the cerebellum in the initial analysis. In addition to the discrepancy in the gene and protein expression pattern observed for the cerebellum samples, we also detected protein expression in both the cingulate cortex and in the hippocampus. These observations did not reflect findings at the transcriptional level. It is possible that the gene is in fact expressed in these regions, however, at such a level that we were unable to detect it. Also, the relationship between mRNA transcript- and protein expression levels was recently demonstrated to be only moderately correlated [30], which could explain the observed differences.

Surprisingly, in a whole genome survey of human tissues, the human ortholog showed exclusive expression in testis. However, expression analysis of *C1orf146* in various areas of the human brain by qRT-PCR revealed expression primarily in samples from the frontal pole. In comparison, very low gene expression was detected in samples from the temporal- and occipital pole. It is possible that *C1orf146* is expressed in a similar restricted area- and layer- specific pattern in the human brain, as observed in rodents. In that case, the mRNA concentration of *C1orf146* might have been too low to be detected in the heterogeneous whole brain samples. In addition, *C1orf146* gene expression was also detected in samples from hippocampus, cerebellum and medulla oblongata, which in part corresponds with areas of observed protein expression in the rat brain.

### LOC689986 might be involved in regulation of gene expression and experience dependent plasticity

*LOC689986* is highly conserved in all vertebrate species, and no orthologous genes could be identified in invertebrates or yeast, indicating that the gene is specific for the vertebrate lineage. The structure and function of the vertebrate CNS is far more complex than the nervous systems in invertebrate species. It is possible that *LOC689986* emerged in a common vertebrate ancestor, exhibiting a specific function related to the increased complexity of the nervous system. The high degree of conservation further suggests an important role for this gene in maintaining a certain functional specialisation (in the brain). However, it is also possible that the gene is involved in different functional specialisations in various vertebrate species, as has been previously demonstrated for the highly conserved gene *Foxp2*[31]. In humans, *FOXP2* has been demonstrated to be important in speech and language development, and similar functional aspects have been observed in songbirds, where the gene seems to be involved in vocal learning. However, in rodents *Foxp2* is apparently involved in synaptic plasticity and motor-skill learning, illustrating the divergence in functional specialisation of this highly conserved gene [31].

In order to explore the possible functional roles of the LOC689986 protein, we analysed the deduced peptide sequence using a range of web-based bioinformatics tools, primarily to search for known functional domains that could link the protein to established protein families. We were not able to identify any known functional domains, regions or sites, nor did we identify any relationship to any established protein families. It is therefore possible that LOC689986 does in fact not have any similarities with other proteins (known or not yet identified). By resolving the LOC689986 protein structure, major structural similarities (folds) might be identified, and could possibly give hints to shared functional roles with proteins of known function.

We also observed that LOC689986 had no predicted cleavage sites, suggesting that it is a non-secretory protein. The fact that the recombinant LOC689986 protein could not be detected in the growth medium of human HeLa cells, supports that the protein was not secreted, at least not when over-expressed in human cell cultures. Neither the recombinant, nor the endogenous protein seemed to be confined to membranes, as both could be detected in the cytosol as well as the nucleus. Moreover, the protein clearly localised to neuronal dendrites, which could indicate a role for the protein in signalling pathways activated in response to an electrical or chemical synapse.

Y2H screens in adult and embryonic mouse brain libraries resulted in the identification of five potential protein-protein interaction partners, common for the two libraries. Chd3, a chromatin remodelling ATPase of the Chromodomain-Helicase-DNA binding family, constitutes the largest component of the Mi-2/NuRD complex [32-35]. It is worth noting that the Y2H screen in embryonic mouse library predicted an interaction to the homologous protein Chd4, which is also a part of the Mi-2/NuRD complex. The complex binds to acetylated histone tails and induces transcriptional repression by chromatin remodelling. The Mi-2/NuRD complex has been shown to contain subunit heterogeneity, where the subunit composition seems to vary with cell type and physiologic signals within a tissue. It has been suggested that incorporation of unique subunits in the Mi-2/NuRD complex might impact the functional specialisation of the complex itself [36]. Should the deduced interaction be validated, LOC689986 could turn out to be a so far unknown unique interaction partner, and might facilitate functional specialisation in distinct areas of the brain, such as the SCx. A potential involvement of LOC689986 in experience dependent plasticity [28] could in fact be linked to chromatin remodelling, and thereby to induction of transcriptional suppression.

## Conclusions

In this study, we have partially characterised a highly conserved gene specific to the vertebrate linage. The gene shows distinct expression patterns in layer 4 of the SCx, including the barrel cortex, and areas of parieto-temporal cortex in rodents. The protein was expressed in SCx, but also in the Purkinje cells of the cerebellar cortex. Furthermore, prediction of potential interaction partners could suggest a role for this protein in control of gene expression, and the functional roles of the protein might involve processing of somatosensory information and experience dependent plasticity. However, the biological function of LOC689986 in the brain and during CNS development remains elusive, and further functional investigation is therefore required.

## Abbreviations

LOC689986: Hypothetical protein LOC689986; FMCx: Frontomedial cortex; TCx: Temporal cortex; OCx: Occipital cortex; qRT-PCR: Quantitative real-time PCR; SCx: Somatosensory cortex; MCx: Motor cortex; C1orf146: Chromosome 1 open reading frame 146; 1700028K03Rik: RIKEN cDNA 1700028K03 gene.

## Competing interests

The authors declare that they have no competing interests.

## Authors’ contributions

Conceived and designed the experiments: KME, BH, CS, VMS. Performed the experiments: KME, JER. Analysed the data: KME, BH, VG, CS, VMS. Wrote the paper: KME, VMS. All authors have read and approved the final manuscript.

## Supplementary Material

Additional file 1 **Cortical tissue dissection.** Consecutive side-by-side tissue samples were extracted from the parietal- temporal- and occipital lobe from the adult rat brain. Tissue samples, covering a matrix of 25 samples, from a total of 6 individual rats were analysed (numbered 1-25). The uppermost row (sample number 21-25) represents samples from the cingulate cortex (arrows indicates the direction). The figure is based upon an image of the adult rat brain (lateral view, image courtesy of Adam C. Puche), acquired from The Olfactory Image Archive. Click here for file

Additional file 2 ***LOC689986 *gene expression in the rat neocortex.** qRT-PCR analysis of the relative *LOC689986* gene expression level in corresponding cortical samples from 6 rats. The relative gene expression level was normalised to the endogenous control *Actb*. Standard error of the mean is indicated for all the samples. x-axis: samples (corresponding to the areas shown in Additional file 1), y-axis: average relative gene expression level, *: samples from five individual rats. Click here for file

Additional file 3 ***In situ *RNA hybridisation analysis of *LOC689986 *gene expression in the brain.** The *LOC689986* gene expression was analysed in representative coronal sections from the whole adult rat brain (20 μm floating sections). The strongest gene expression was observed in layer 4 of the somatosensory cortex. **A**. Bregma 0.48 mm, **B**. Bregma -0.24 mm, **C**. Bregma -1.72. The areas shown in **A**, **B** and **C** correspond to regions illustrated in the schematic drawing of the adult rat brain (drawing was obtained from motifolio.co). Click here for file

Additional file 4 **Expression pattern of the human orthologous gene *C1orf146*.** qRT-PCR analysis of the relative *C1orf146* gene expression level in tissue samples from 8 different human brain regions (x-axis). The gene expression of *C1orf146* was normalised against the endogenous control *ARBP*. The relative gene expression level is indicated on the y-axis. Ct values are listed below. Click here for file

Additional file 5 **Microarray gene expression pattern of *C1orf146 *in 32 different human tissue samples.** The samples are from the Tissue Gene Expression Database (Human Body Map, Applied Biosystems), and are listed on the x-axis. y-axis: normalised signal intensity. Click here for file

Additional file 6 **LOC689986 is located in astrocytes.** Confocal laser scanning images of a section from piriform cortex that was double labelled for LOC689986 (**A**, green) and the astrocytic marker glutamine synthetase (**B**, purple). The overlay (**C**) shows that astrocytes are labelled for LOC689986 (white). Arrows in **A** and **B** highlight LOC689986 positive astrocytes. The inset shows a double labelled astrocyte at higher magnification. Click here for file
