# Supplementary figures and images for: LOC689986, a unique gene showing specific expression in restricted areas of the rodent neocortex
Source: BMC Neurosci. 2013 Jul 11;14:68. doi: 10.1186/1471-2202-14-68 (PMC3717020; doi:10.1186/1471-2202-14-68)

Additional file 1

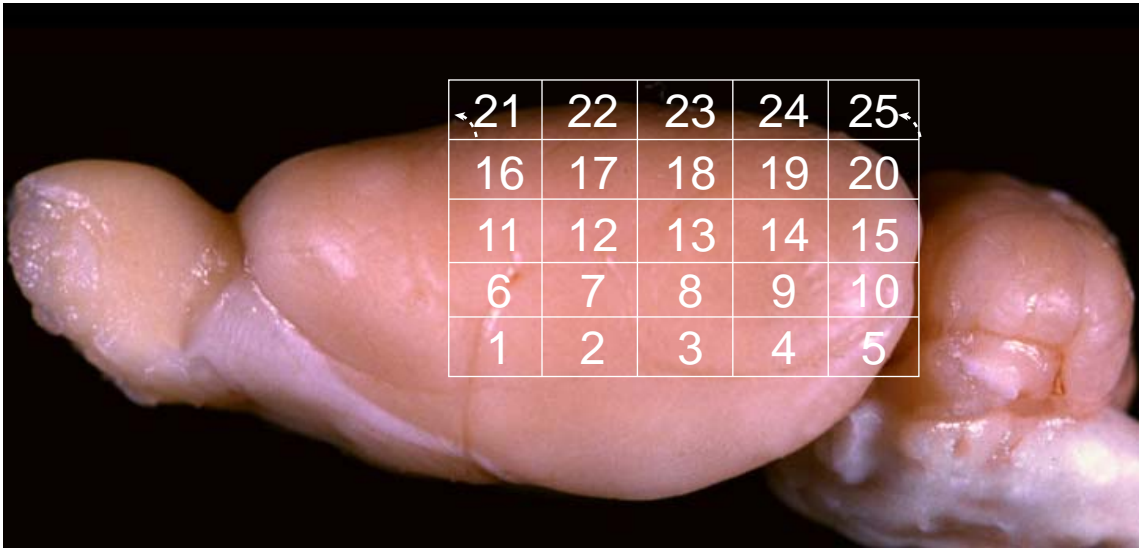

Supplement: Additional file 1 — Cortical tissue dissection. Consecutive side-by-side tissue samples were extracted from the parietal- temporal- and occipital lobe from the adult rat brain. Tissue samples, covering a matrix of 25 samples, from a total of 6 individual rats were analysed (numbered 1-25). The uppermost row (sample number 21-25) represents samples from the cingulate cortex (arrows indicates the direction). The figure is based upon an image of the adult rat brain (lateral view, image courtesy of Adam C. Puche), acquired from The Olfactory Image Archive. [file 1471-2202-14-68-S1.pdf]

Additional file 2

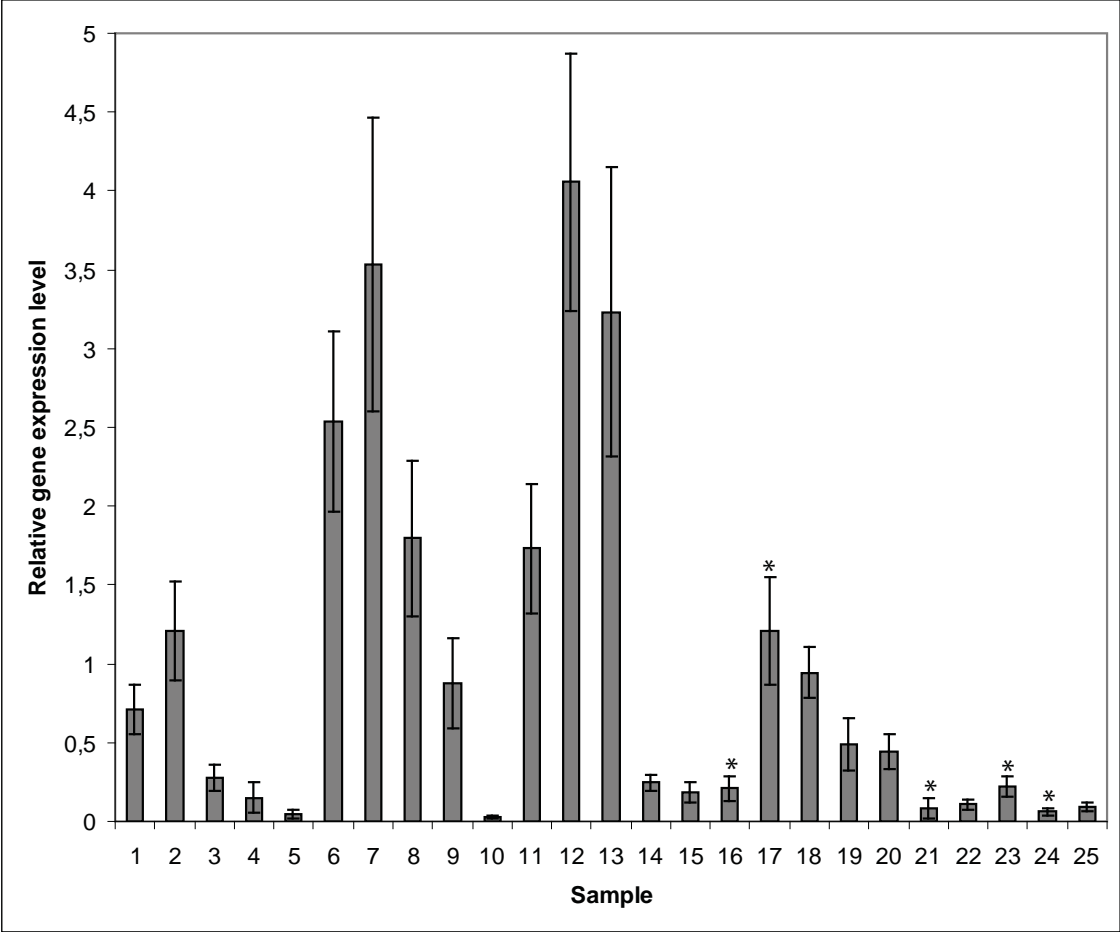

Supplement: Additional file 2 — LOC689986 gene expression in the rat neocortex. qRT-PCR analysis of the relative LOC689986 gene expression level in corresponding cortical samples from 6 rats. The relative gene expression level was normalised to the endogenous control Actb. Standard error of the mean is indicated for all the samples. x-axis: samples (corresponding to the areas shown in Additional file 1), y-axis: average relative gene expression level, *: samples from five individual rats. [file 1471-2202-14-68-S2.pdf]

Additional file 3

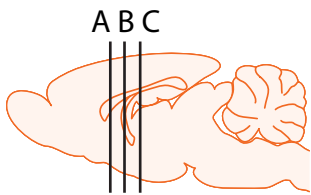

**A**

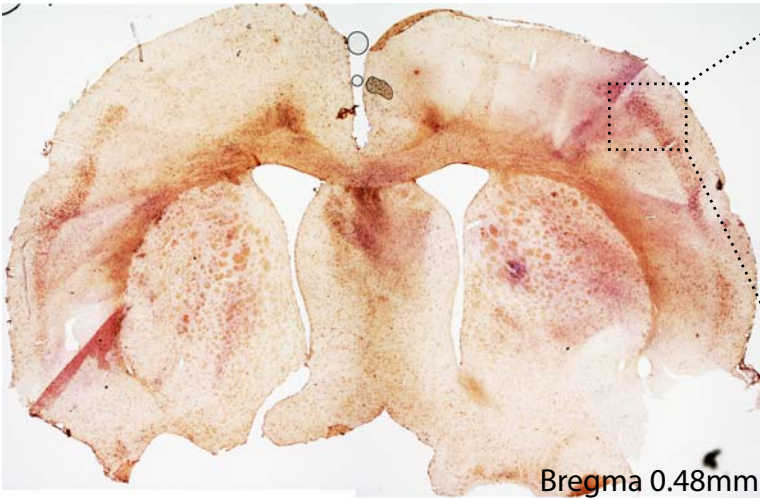

Bregma 0.48mm

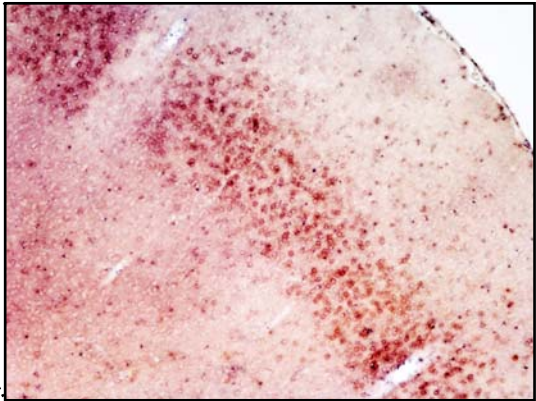

**B**

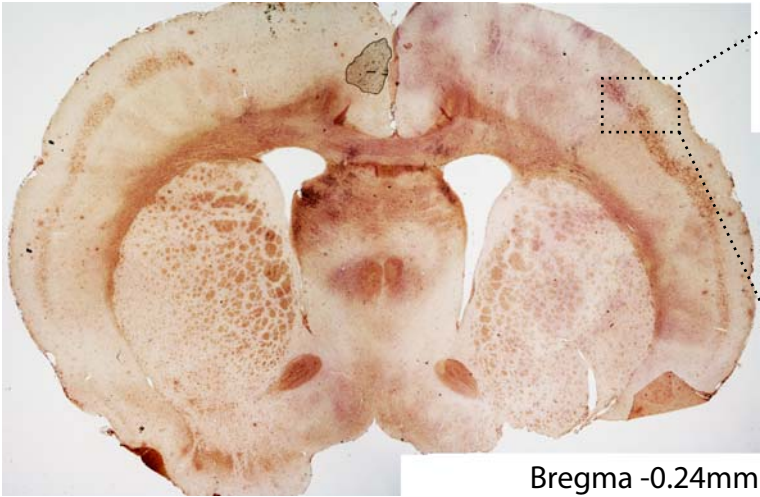

Bregma -0.24mm

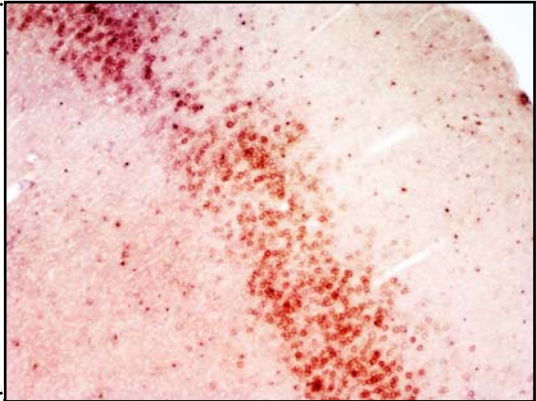

**C**

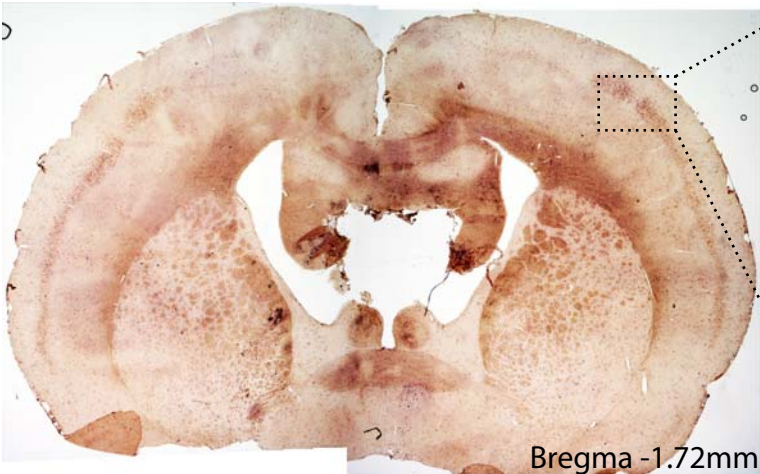

Bregma -1.72mm

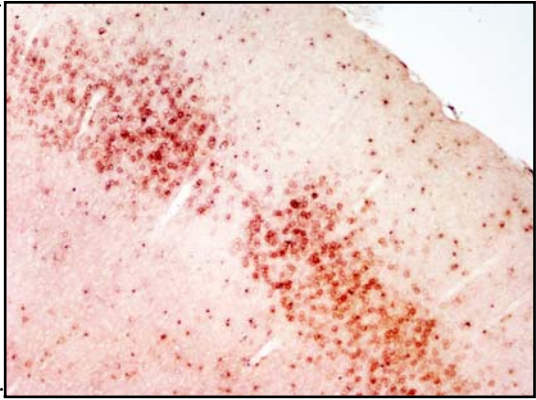

Supplement: Additional file 3 — In situ RNA hybridisation analysis of LOC689986 gene expression in the brain. The LOC689986 gene expression was analysed in representative coronal sections from the whole adult rat brain (20 μm floating sections). The strongest gene expression was observed in layer 4 of the somatosensory cortex. A. Bregma 0.48 mm, B. Bregma -0.24 mm, C. Bregma -1.72. The areas shown in A, B and C correspond to regions illustrated in the schematic drawing of the adult rat brain (drawing was obtained from motifolio.co). [file 1471-2202-14-68-S3.pdf]

Additional file 5

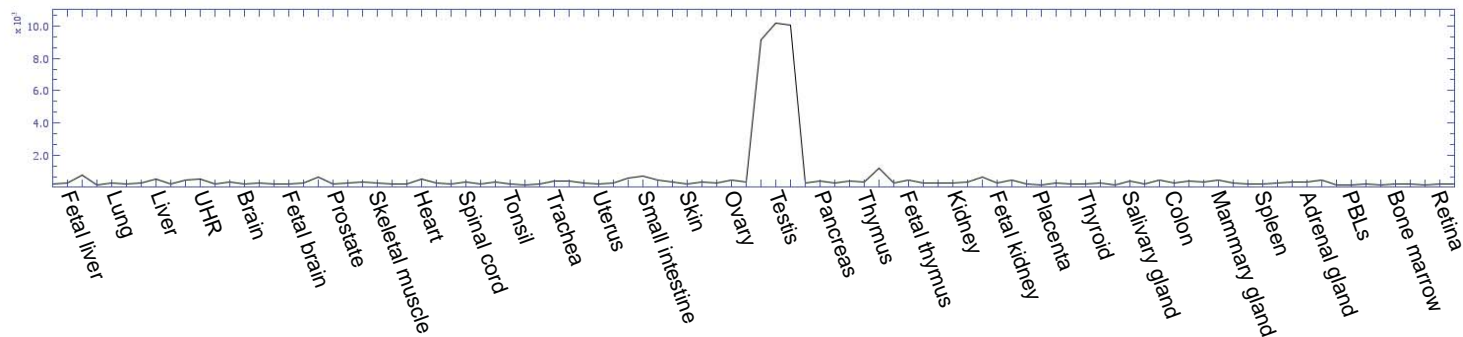

Supplement: Additional file 5 — Microarray gene expression pattern of C1orf146 in 32 different human tissue samples. The samples are from the Tissue Gene Expression Database (Human Body Map, Applied Biosystems), and are listed on the x-axis. y-axis: normalised signal intensity. [file 1471-2202-14-68-S5.pdf]

## Additional file 6

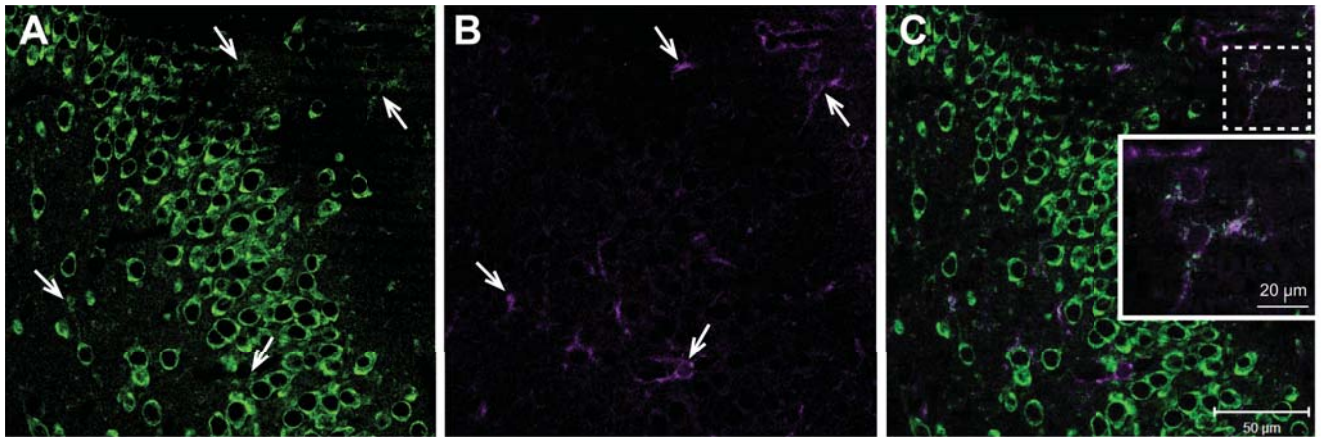

Supplement: Additional file 6 — LOC689986 is located in astrocytes. Confocal laser scanning images of a section from piriform cortex that was double labelled for LOC689986 (A, green) and the astrocytic marker glutamine synthetase (B, purple). The overlay (C) shows that astrocytes are labelled for LOC689986 (white). Arrows in A and B highlight LOC689986 positive astrocytes. The inset shows a double labelled astrocyte at higher magnification. [file 1471-2202-14-68-S6.pdf]
