# Supplementary material for: LOC689986, a unique gene showing specific expression in restricted areas of the rodent neocortex
Source: BMC Neurosci. 2013 Jul 11;14:68. doi: 10.1186/1471-2202-14-68 (PMC3717020; doi:10.1186/1471-2202-14-68)
Supplement: Additional file 4 — Expression pattern of the human orthologous gene C1orf146. qRT-PCR analysis of the relative C1orf146 gene expression level in tissue samples from 8 different human brain regions (x-axis). The gene expression of C1orf146 was normalised against the endogenous control ARBP. The relative gene expression level is indicated on the y-axis. Ct values are listed below. [file 1471-2202-14-68-S4.pdf]

Additional file 4

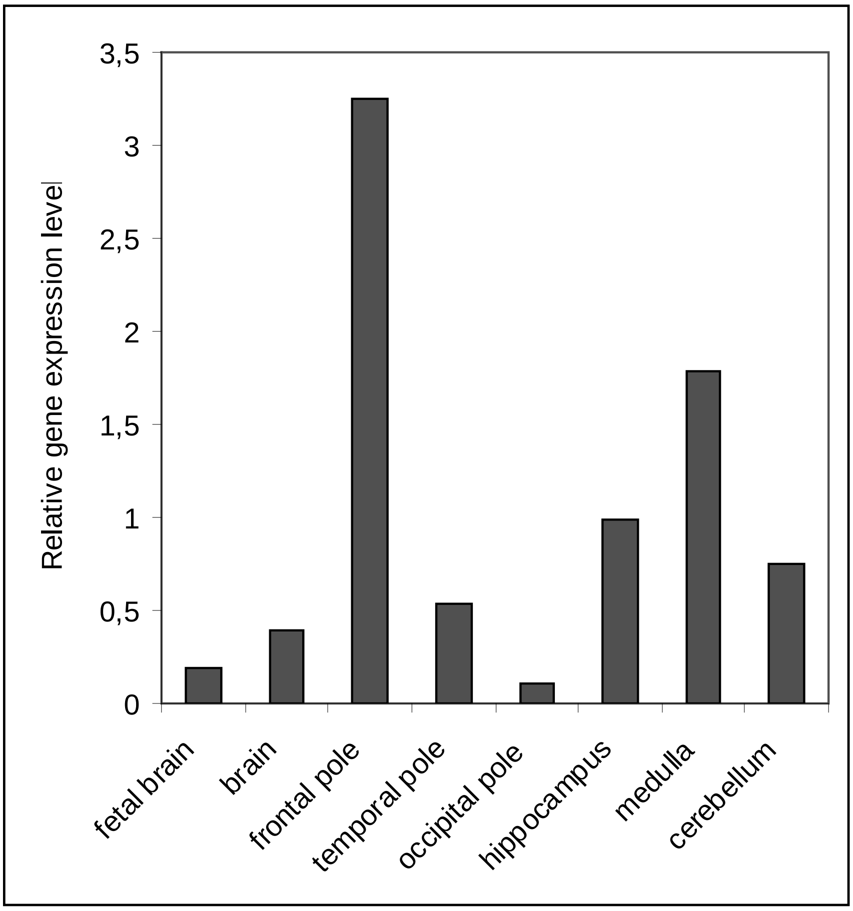

	**Ct Mean values**	
**Sample**	*C1orf146*	*ARBP*
fetal brain	36,1	20,5
brain	36,9	22,3
frontal pole	34,5	23,0
temporal pole	37,5	23,4
occipital pole	39,8	23,3
hippocampus	36,5	23,3
medulla	35,8	23,5
cerebellum	36,2	22,6
